# Association of Gestational Hypertensive Disorders with Retinopathy of prematurity: A Systematic Review and Meta-analysis

**DOI:** 10.1038/srep30732

**Published:** 2016-08-05

**Authors:** Priscilla Y. L. Chan, Shu-Min Tang, Sunny C. L. Au, Shi-Song Rong, Henry H. W. Lau, Simon T. C. Ko, Danny S. C. Ng, Li Jia Chen, Jason C. S. Yam

**Affiliations:** 1 Department of Ophthalmology and Visual Sciences, The Chinese University of Hong Kong, Hong Kong; 2 Department of Ophthalmology, Tung Wah Eastern Hospital, Hong Kong

## Abstract

The role of gestational hypertensive disorders, which includes both pre-eclampsia and gestational hypertension, in the development of retinopathy of prematurity (ROP) has been controversial. Therefore, this systematic review and meta-analysis is to evaluate the association between gestational hypertensive disoders and ROP. Eligible studies published up to June 5, 2016 were identified from MEDLINE and EMBASE that evaluated the association between the two conditions. Totally 1142 published records were retrieved for screening, 925 of them eligible for detailed evaluation. Finally 19 studies involving 45281 infants with 5388 cases of ROP met our criteria for meta-analysis. Gestational hypertensive disorders were not associated with ROP (unadjusted OR: 0.89; P = 0.38; adjusted OR: 1.35; P = 0.18). Subgroup analyses also revealed no significant association between ROP with pre-eclampsia (unadjusted OR: 0.85; P = 0.29; adjusted OR:1.29; P = 0.28) or with gestational hypertension (unadjusted OR: 1.10; P = 0.39; adjusted OR: 1.25; P = 0.60) separately. Sensitivity analysis indicated our results were robust. We concluded no significant association between gestational hypertensive disorders and ROP. More large scale well-conducted prospective cohorts on the topic are needed.

Retinopathy of prematurity (ROP) is a retinal vascular disease characterized by abnormal vascular development in the retinas of premature infants. It is a leading cause of childhood blindness despite current surgical and laser treatment^1^. Given its high prevalence with significant morbidity, the identification of risk factors together with effective prevention and timely treatment are essential for preserving lifelong vision in these neonates. Important risk factors identified so far include low gestational age, low birth weight, supplementary oxygen, neonatal sepsis, intraventricular hemorrhage, hyperglycemia etc^2^. During the developmental process, retinal blood vessels grow outwards from the centre of the retina and the process is completed a few weeks before the normal time of delivery^3^. In preterm infants, the exposure to high oxygen concentrations reduces the retinal levels of proangiogenic growth factors including vascular endothelial growth factor (VEGF), leading to cessation of blood vessels growth^3^. Subsequently, relative hypoxia leads to stimulation of higher VEGF levels causing abnormal overgrowth of the retinal vasculature. These abnormal blood vessels may bleed and the blood together with the abnormal vasculature when reabsorbed, will cause traction to the retina leading to retinal detachment and early blindness^3^.

Gestational hypertensive disorders encompass a spectrum of disorders ranging from gestational hypertension, pre-eclampsia to full-blown eclampsia where the lives of both the mother and fetus are threatened^4^. Pre-eclampsia (PET) is a disorder during mid- to late-pregnancy characterized by high blood pressure and damage to another organ most commonly the kidneys^4^. It is diagnosed when there is a persistent hypertension with proteinuria in a pregnant woman beyond 20 weeks of pregnancy^4^. PET when severe can lead to significant prematurity which in turn affects neonatal outcomes due to the severity of prematurity. However, pre-clampsia itself has been shown to have controversial effects on ROP. Certain individual studies have shown that PET is protective over ROP, possibly due to the oxidative stress exerted on the fetal development^5^,^6^,^7^. In addition, Yu *et al.* specifically included comparisons between gestational hypertension and PET with ROP and it concluded that PET, but not gestational hypertension, was associated with a reduced risk of ROP in preterm births^5^. On the other hand, some found PET to be a risk factor for developing ROP^8^,^9^,^10^ due to the ischemic and angiogenic stress on retinal vascularization whilst others concluded no significant association^11^,^12^. This disagreement may be due to a relatively small sample size, lack of control for known risk factors, wide variation in outcome measures as well as the lack of clear definition of gestational hypertensive disorders. In an effort to resolve the discrepancy observed across studies, we conducted a systematic review and meta-analysis to synthesize the literature that measures the association of gestational hypertensive disorders with ROP.

## Methods

### Searching Strategy

Online databases, EMBASE and MEDLINE (Medical Literature Analysis and Retrieval System Online, via Ovid platform), were used for electronic search from their starting date to June 5, 2016. Both controlled vocabularies and free words, such as terms “RETINOPATHY OF PREMATURITY, ROP”, and “PREECLAMPSIA, ECLAMPSIA AND PREECLAMPSIA, GESTSATIONAL HYPERTENSION, MATERNAL HYPERTENSION, RETROLENTAL FIBROPLASIA, NEONATAL OUTCOME, NEURODEVELOPMENT OUTCOME, PRETERM OUTCOME” were used in our search. Detailed searching strategies is given in Supplementary Table 1. All articles and abstracts published in English were identified. The citation lists of relevant articles and reviews were screened to identify additional eligible articles which might have been missed by electronic search.

### Study selection

The inclusion criteria were as follows (1) a cross-sectional, prospective cohort or case-control study which evaluated the association between ROP and gestational hypertensive disorders (i.e., gestational hypertension, PET and eclampsia); (2) studies which reported the outcomes, such as odds ratio (OR) or risk ratio (RR) and their confidence intervals (CIs), or numerical counts that allow the calculation of the aforementioned outcomes. Animal studies, case reports, reviews, abstracts, conference proceedings, editorials, and studies with insufficient data or inconsistent outcomes for meta-analysis were excluded. Only human studies on clinical aspects of ROP published in English were included.

The studies were grouped into three groups for comparison: pre-eclampsia as exposure only; gestational hypertension as exposure only; and overall pre-eclampsia and gestational hypertension included as exposure. A summary of the studies is shown in Table 1.

### Data Extraction

According to the Meta-analysis of Observational Studies in Epidemiology (MOOSE) guidelines (Supplementary Table 2) for reporting meta-analysis of observational studies, all retrieved records from individual studies were screened and reviewed by two independent investigators (PYLC and SMT)^13^. Data were extracted with customized data sheets. Discrepancies were resolved through discussion between the two reviewers and a third reviewer (JCSY). Data collected included: PubMed ID (if available), year of publication, first author, design and location of study, ethnicity, definition of pre-eclampsia or gestational hypertension and ROP, sample size, and association results (i.e., mean and standard deviation).

### Quality Assessment

We assessed the methodological quality using the Newcastle-Ottawa Scale (NOS, available in the public domain at http://www.ohri.ca/programs/clinical_epidemiology/oxford.asp) for case-control or cohort studies as appropriate^14^. NOS contains three demensions, i.e., potential selection bias, comparability and ascertainment of exposure. We assigned 1 star for birth weight and 1 star for gestational age when assessing the comparability. The NOS has a maximum score of 9 stars. A score of 5 or above is considered as having satisfactory quality in this study^15^. Two reviewers (PYLC and SCLA) independently assessed the quality of each study. Discrepancies were resolved through discussion between the two reviewers and a third reviewer (SMT).

### Statistical Analysis

The studies were grouped and analyzed by type of exposures, including (1) overall PET and gestational hypertension; (2) PET; and (3) gestational hypertension. Using the RevMan5 software, we inputted all unadjusted numerical counts that we could gather to calculate each study’s individual univariate odds ratio (OR) whenever available. As the adjusted ORs and 95% CI were more accurate to estimate true associations, if studies have provided multivariate ORs using factors they identified for adjustment, we included them in a separate comparison and meta-analyzed. This was done for all three groups of studies.

We calculated pooled odd ratio (OR) comparing risk of ROP among infants born to women with gestational hypertension/pre-eclampsia to those without it using both a random effects model and a fixed effects model. Heterogeneity between studies was evaluated using Q and I^2^ statistics. I^2^ is the amount of total variation that is due to variation between studies. I^2^ values of approximately 25%, 50% and 75% indicate low, moderate and high heterogeneity, respectively. If P for Q < 0.1 or I^2^ > 50%, a random-effects model (DerSimonian and Laird method) was used^16^, otherwise we used a fixed-effects model (Mantel-Haenszel method)^17^. Publication bias was assessed using Egger’s test, where a value of p < 0.05 was considered statistically significant^18^,^19^. We conducted sensitivity analyses excluding one study at a time to test whether the results were robust. Subgroup analyses were done for different exposures i.e., PET only, gestational hypertension only and overall PET and gestational hypertension. One subgroup analysis was also allocated for the effect of PET on the severity of ROP.

## Results

### Description of the Studies

**Figure 1:**
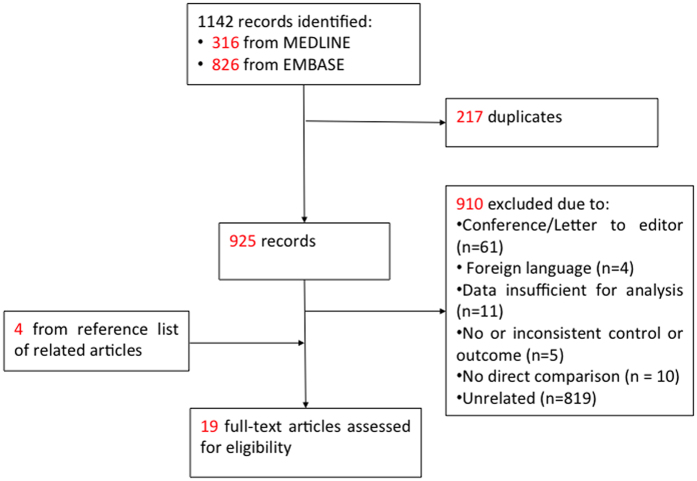
Study flow diagram.

A total of 1142 potentially relevant studies were yielded from the literature search. Among them, 19 studies^5^,^6^,^8^,^9^,^10^,^11^,^12^,^20^,^21^,^22^,^23^,^24^,^25^,^26^,^27^,^28^,^29^,^30^,^31^ were identified as eligible for meta-analysis (Fig. 1), involving 45,281 infants with 5388 cases of ROP. Ten of them were prospective cohort^6^,^9^,^20^,^21^,^22^,^24^,^27^,^28^,^29^,^30^ while the remaining were retrospective case-control studies^5^,^8^,^10^,^11^,^12^,^23^,^25^,^26^,^31^. A summary of the studies is shown in Table 1. Among them, 14 studies studied infants born specifically to mothers with PET only^6^,^8^,^9^,^10^,^11^,^20^,^21^,^23^,^25^,^26^,^28^,^29^,^30^,^31^, while 4 studied those born to mothers with gestational hypertension^12^,^22^,^24^,^27^. There was one study which included separate data of both PET and gestational hypertension as the exposure^5^. Studies were categorized as “PET only” (n = 15), “gestational hypertension only” (n = 5) and “overall PET and gestational hypertension” for our analyses (n = 19). (Table 1).

All studies provided either numerical counts, unadjusted or adjusted ORs to measure the association between ROP and pre-eclampsia/gestational hypertension. The objectives of the studies varied. In 14 studies, risk factors for developing ROP were evaluated and gestational hypertesive disorders was included as one of the factors to be studied^8^,^10^,^11^,^21^,^22^,^23^,^24^,^25^,^26^,^27^,^28^,^29^,^30^,^31^. The remaining 5 studies specifically evaluated the association between pre-eclampsia/gestational hypertension and ROP^5^,^6^,^9^,^12^,^20^. Clinical definition of pre-eclampsia was only provided in 5 studies^5^,^9^,^20^,^21^,^26^. The mean gestational age of all eligible studies ranged between 26 to 32 weeks. On assessment of the methodological quality using the NOS, all studies attain the score 5 or above (Table 1). ROP was mostly defined as per the International Classification for Retinopathy of Prematurity. Six studies looked at the severity of ROP (treatable ROP at stage 3 or above)^6^,^8^,^9^,^10^,^26^,^31^.

### Comparison of overall gestational hypertension + PET on ROP

#### Univariate comparison

**Figure 2:**
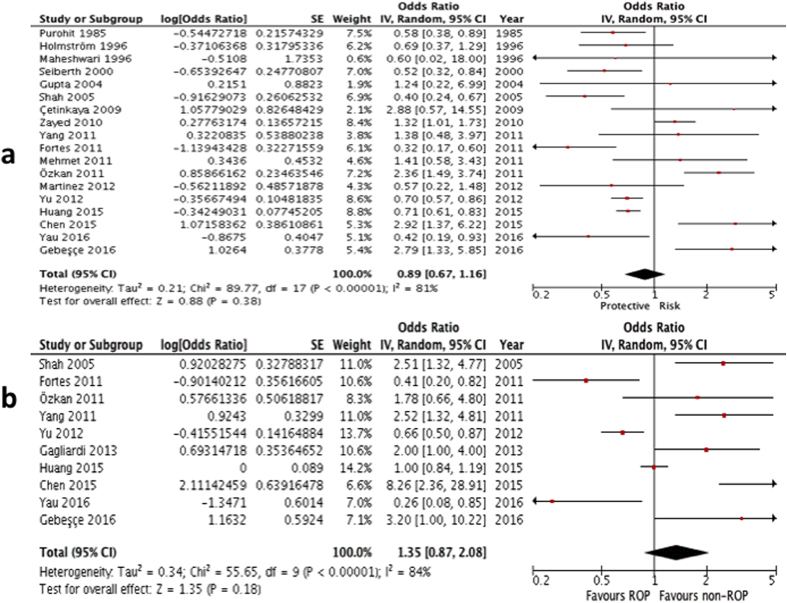
(**a**) Forest plot for univariate analysis of 18 studies examining the effect of overall gestational hypertensive disorder on ROP (any type). The bars with squares in the middle represent 95% confidence intervals (95% CIs) and odds ratios (ORs). The central vertical solid line indicates the ORs for null hypothesis. (**b)** Forest plot for multivariate analysis of 10 studies examining the effect of overall gestational hypertensive disorder on ROP (any type). The bars with squares in the middle represent 95% confidence intervals (95% CIs) and odds ratios (ORs). The central vertical solid line indicates the ORs for null hypothesis.

Sixteen^5^,^6^,^8^,^9^,^10^,^11^,^12^,^20^,^23^,^24^,^25^,^26^,^27^,^28^,^29^,^30^ out of the 19 studies (except Gagliardi *et al.*, Yau *et al.* and Chen *et al.*) provided numerical counts on the comparison between the two variables, and therefore their unadjustated ORs were calculated. For the studies by Chen *et al.*^21^ and Yau *et al.*^31^, only unadjusted ORs were provided, but not the numerical counts of ROP and gestational hypertensive disorders. Gagliardi *et al.* only provided adjusted OR^22^. The unadjusted ORs from the above 18 studies (except Gaglidardi *et al.*^22^) were then put into inverse variance formula and an analysis on their univariate ORs were performed. It showed no significant association. (summary OR: 0.89; 95% CI: 0.67 – 1.16, P = 0.38; I^2^ = 81%; Fig. 2a; Table 2).

#### Multivariate comparison

Ten out of the 19 studies provided an adjusted OR, hence they were included for the multivariate comparison^5^,^6^,^8^,^9^,^10^,^22^,^23^,^26^,^31^,^32^. Analysis on their multivariate ORs also yielded no significant association (summary OR: 1.35; 95% CI: 0.87 – 2.08, P = 0.18; I^2^ = 84%,; Fig. 2b; Table 2).

### Subgroup analyses

#### Association between PET and ROP

**Figure 3:**
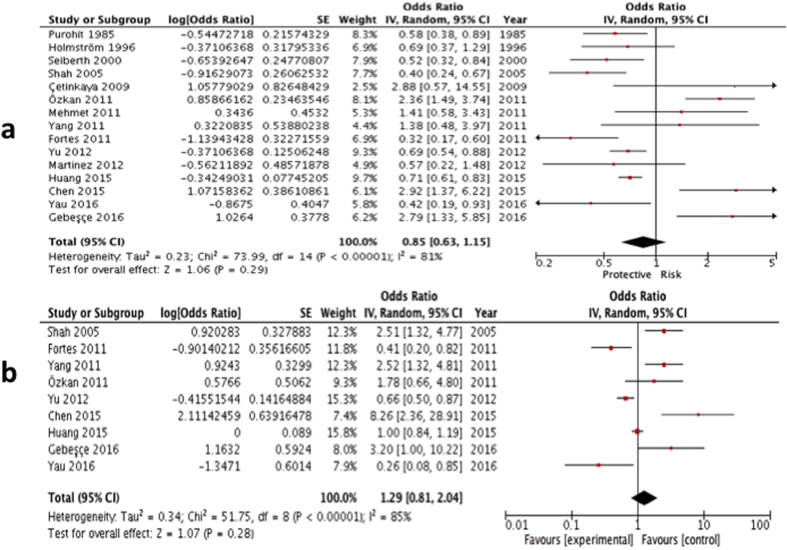
(**a**) Forest plot for univariate analysis of 15 studies examining the effect of PET only exposure on ROP (any type). The bars with squares in the middle represent 95% confidence intervals (95% CIs) and odds ratios (ORs). The central vertical solid line indicates the ORs for null hypothesis. (**b**) Forest plot for multivariate analysis of 9 studies examining the effect of PET only exposure on ROP (any type). The bars with squares in the middle represent 95% confidence intervals (95% CIs) and odds ratios (ORs). The central vertical solid line indicates the ORs for null hypothesis.

Univariate comparison: Thirteen studies provided numerical counts on the comparison between PET and ROP^5^,^6^,^8^,^9^,^10^,^11^,^20^,^23^,^25^,^26^,^28^,^29^,^30^, while Yau *et al.* and Chen *et al.* provided unadjusted ORs on the two conditions only^21^,^31^. Similar to the comparison of overall gestational hypertensive disorder, all the unadjusted ORs of the above 15 studies were put into invariance variance formula to generate this univariate comparison. Analysis on their univariate ORs showed no significant association (summary OR: 0.85; 95% CI: 0.63 – 1.15, P = 0.29; I^2^ = 81; Fig. 3a; Table 2).

Multivariate comparison: Nine studies provided an adjusted OR, hence they were included for the multivariate comparison^5^,^6^,^8^,^9^,^10^,^21^,^23^,^26^,^31^. Analysis on their multivariate ORs, however, yielded no significant association between PET and ROP (summary OR: 1.29; 95% CI: 0.81–2.04, P = 0.28; I^2^ = 85; Fig. 3b; Table 2).

#### Association between gestational hypertension and ROP

**Figure 4:**
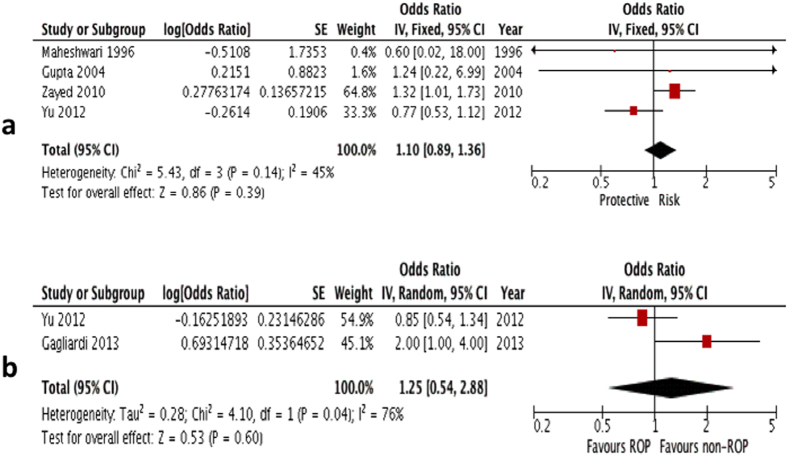
(**a**) Forest plot for univariate analysis of 4 studies examining the effect of gestational hypertension only exposure on ROP (any type). The bars with squares in the middle represent 95% confidence intervals (95% CIs) and odds ratios (ORs). The central vertical solid line indicates the ORs for null hypothesis. (**b)** Forest plot for multivariate analysis of 2 studies examining the effect of gestational hypertension only exposure on ROP (any type). The bars with squares in the middle represent 95% confidence intervals (95% CIs) and odds ratios (ORs). The central vertical solid line indicates the ORs for null hypothesis.

Univariate comparison: Four studies were included in the univariate comparison. Analysis on their univariate ORs showed no significance (summary OR: 1.10; 95% CI: 0.89–1.36, P = 0.39; I^2^ = 45; Fig. 4a; Table 2)^5^,^12^,^24^,^27^.

Multivariate comparison: Two studies provided an adjusted OR, hence they were included for the multivariate comparison^5^,^22^. Analysis on their multivariate ORs also yielded no significant association (summary OR: 1.25; 95% CI: 0.54–2.88, P = 0.60; I^2^ = 76; Fig. 4b; Table 2).

#### Analysis based on severity of ROP

**Figure 5:**
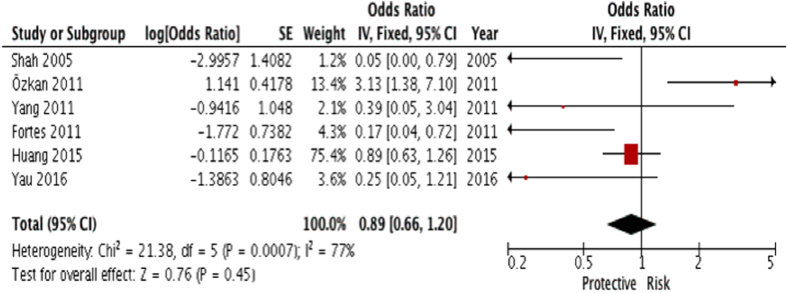
Forest plot for crude data analysis of 6 studies examining the effect of overall gestational hypertensive disorder on severity of ROP (stage 3 or above).

Severe ROP was defined as stage 3 ROP or above^33^,^34^. Four studies provided raw data on the severity of ROP from either PET, gestational hypertension or both^6^,^8^,^9^,^10^ while 2 provided univariate ORs, hence the univariate comparison was used for analysis^26^,^31^. These data were input to calculate for any significant association. The results showed no significance. (summary OR: 0.89; 95% CI: 0.66–1.20, P = 0.45; I^2^ = 77; Fig. 5; Table 2).

### Publication bias and sensitivity analysis

Most of the included studies had a robust design and, therefore, had low risk for introduction of bias (Table 1). The Egger’s test didn’t show any publication bias (Table 2). Subsequently, a sensitivity analysis was performed. We performed the analyses by sequentially omitting one study at a time to confirm the results. The heterogeneity and results didn’t change in the sensitivity analysis.

## Discussion

This present systematic review and meta-analysis of both unadjusted data and adjusted data showed no significant correlation between gestational hypertensive disorders (both PET and gestational hypertension included) and the development of ROP. The included studies were of good quality according to the Newcastle-Ottawa Scale as mentioned in Quality Assessment. To our knowledge, this meta-analysis is the first of its kind that analyzes the effect of perinatal disease in the form of gestational hypertensive disorders on the development of ROP in infants.

How gestational hypertensive disorders affects ROP is controversial and postulated to be of different mechanisms. Most discussions surround on the level of maternal proangiogenic factors (VEGF) which are induced by hypoxia and the oxidative stress infants born to mothers with gestational hypertensive disorders are exposed to^5^,^6^,^7^,^11^,^12^.

Kulkarni *et al.* reported a lower plasma VEGF and placental growth factor levels with a higher umbilical cord VEGF levels^35^. The dysregulation of the proangiogenic factors in pre-eclmpasia along with maternal oxidative stress and placental ischemia have been suggested to cause retinal hypoxia and elevation of VEGF in infants born to mothers with gestational hypertensive disorders^35^. Another possible mechanism responsible, proposed by Ozkan *et al.* is that the increased oxidative stress along with the increase of pro-inflammatory cytokine levels in infants born to PET mothers may interfere with the normal retinal vascularization in vulnerable retinas^36^.

However, it has also been reported that the level of sFlt1, a VEGF inhibitor which can bind VEGF and prevent it from signaling through its receptors together wtih another antiangiogenic factor soluble endoglin (sEng) were markedly elevated in pre-eclamptic mothers^37^,^38^. Hence Yu *et al.* proposed several mechanisms in which infants born to pre-eclamptic mothers might be exposed to the higher antiangiogenic factors level (sFlt1, sEng)^5^. First, the fetal placenta and retina might produce more antiangiogenic factors in response to the hypoxia since hypoxia is important in the pathogenesis of both pre-eclampsia and ROP. Second, the antiangiogenic factors might cross through the placenta to enter the fetal cirulation^5^. However, this has been proven against by some clinical studies^39^,^40^. Third, the fetus might be exposed to antiangiogenic factors via the amniotic fluid which has been proven to be a rich source of sFlt1 and sEng^41^.

Since both our univariate and multivariate comparison did not reveal a consistent result, the conclusion remains inconclusive and further studies elucidating the mechanisms of the finding in this study will be needed to foster better understsanding and come to a clinical conclusion for the approach on ROP in infants born to mothers with gestational hypertensive disorders.

One major limitation in our study is the heterogeniety of the studies involved as demonstrated by the I^2^ value. We explored heterogeneity by grouping the studies by type of gestational hypertensive disorders i.e. PET and gestational hypertension, but heterogeneity remained substantial among all the subgroups. One important source of heterogeneity may be the substantial variation in the definition of gestational hypertensive disorders in different countries. The definitions of gestsational hypertensive disorders may be different (which most studies did not provide). Races and ethnicity may also influence the predisposition to developing ROP. Also, a significant proportion of studies that included data with gestational hypertensive disorders and ROP were retrospective studies. As illustrated in the summary table, seven studies provided prospective studies on PET^6^,^9^,^20^,^21^,^28^,^29^,^30^ and three on gestational hypertensive disorders^22^,^24^,^27^.

Ozkan *et al.* which concluded that PET increased ROP development in premature infants suggested the angiogenic and oxidative stress inflammatory model to explain its results^9^. They, however, did not mention any limitations to their studies. The number of cases included in their study was also small, 385 infants. Shah *et al.*^8^ concluded that PET increased ROP development among very low birth weight infants based on their multiple regression (2.51). However, using their numerical counts, the calculated odds ratio of 0.40 is conindentally the reciprocal of the result from multiple regression, giving rise to a conflicting result. Zayed *et al.*^12^ is the study which is the largest cohort out of all studies included. However, it only studied the association between maternal gestsational hypertension and the development of ROP, without including those with PET.

## Conclusion

We concluded that through our comprehensive analysis using both univariate and multivariate comparisons between the two common types of gestational hypertensive disorders and the development of ROP, there is no significant association between the two based on current evidence. Hence, gestational hypertensive disorders cannot be definitely considered as a protective or risk factor for ROP based on current evidence. Further well-designed large scale prospective cohort specifically evaluating the two conditions may be needed in order to better evaluate the causal relationship between gestational hypertensive disorders and ROP.

## Additional Information

**How to cite this article**: Chan, P. Y. L. *et al.* Association of Gestational Hypertensive Disorders with Retinopathy of prematurity: A Systematic Review and Meta-analysis. *Sci. Rep.* **6**, 30732; doi: 10.1038/srep30732 (2016).

## Supplementary Material

Supplementary Information

## Figures and Tables

**Table 1:** Characteristics osf studies included for the meta-analysis.

First author	Year	Ethnicity	Study design	Phenotype of Maternal Hypertension	Sample size		Adjusted OR (95%CI)	Factors adjustd	Results of ROP with maternal hypertension	NOS qualityanalysis
					ROP	No ROP				
Purohit, D. M.	1985	USA	Prospective cohort	Toxemia	328	2697	N/A	N/A	Increased risk of retrolental fibroplasia	7
Holmström, G.	1996	Stockholm, Sweden	Retrospective case-control	Pre-eclampsia	81	121	N/A	N/A	Inconclusive	5
Maheshwari, R.	1996	New Delhi, India	Prospective cohort	Maternal pregnancy-induced hypertension	13	53	N/A	N/A	Inconclusive	8
Seiberth, V.	2000	Germany	Retrospective case-control	Maternal pre-eclampsia	145	257	N/A	N/A	Inconclusive	5
Gupta, V. P.	2004	New Delhi, India	Prospective cohort	Maternal hypertension	13	47	N/A	N/A	No association	8
Shah, V. A.	2005	Singapore	Retrospective case-control	Maternal pre-eclampsia	165	399	2.51 (1.32–4.7)	Pulmonary haemorrhage, duration of mechanical ventilation, duration of CPAP, BW	Predictive of ROP	6
Çetinkaya, M.	2009	Bursa, Turkey	Prospective cohort	Pre-eclampsia	10	74	N/A	N/A	No association	9
Zayed, M. A.	2010	North Carolina, USA	Restrospective case-control	Maternal gestational hypertension	322	4818	N/A	N/A	No association	6
Fortes Filho, J. B.	2011	Brazil	Prospective cohort	Maternal pre-eclampsia	97	227	0.406 (0.202–0.817)	Gestational age (GA), antenatal steroid treatment, use of oxygen in mechanical ventilation, use of indomethacin, blood transfusion, vaginal delivery, small for gestational age (SGA)	Lowers risk of ROP in very-low-birth-weight (VLBW) infants	8
Özkan, H.	2011	Bursa, Turkey	Prospective cohort	Maternal pre-eclampsia	109	276	1.78 (0.66–1.90)	GA, birth weight (BW), duration of mechanical ventilation, duration of total oxygen	Increased risk of ROP in premature infants	8
Yang, C. Y.	2011	Northern Taiwan	Retrospective case-control	Maternal pre-eclampsia	99	117	2.52 (1.32–4.7)	Duration of mechanical ventilation and BW	Predictive of ROP	5
Mehmet, S.	2011	Izmir, Turkey	Prospective cohort	Maternal pre-eclampsia	86	117	N/A	N/A	Inconclusive	8
Chen, Y.	2011	North and South China	Prospective cohort	Pre-eclampsia	N/A	N/A	8.26 (2.36–28.9)	GA, BW, maternal supplemental oxygen adminstration, fetus number	Inconclusive	9
Martinez-Cruz, C. F.	2012	Mexican City	Prospective cohort	Maternal pre-eclampsia	34	105	N/A	N/A	Not mentioned	7
Yu, X. D.	2012	USA	Retrospective case-control	Pre-eclampsia (PET) and gestational hypertension (HTN)	1053	24420	0.66 (0.50–0.87)	GA, mode of delivery, number of fetuses, race, body-mass-index at delivery, BW, gender, blood transfusion, congenital anomalies and intraventricular haemorrhage (IVH)	PET but not gestational HTN lowers risk of ROP in preterm births	9
Ggaliardi, L.	2013	Italy	Prospective cohort	Gestational hypertensive disorder	N/A	N/A	2 (1–4)	Level of birth centre and GA	Not mentioned	7
Huang, H. C.	2015	Taiwan	Retrospective review	Maternal pre-eclampsia	2785	2933	1 (0.84–1.20)	GA, BW, Cesarean section, sex, SGA, Apgar score at 5 min, RDS, transfusion, PDA, sepsis	No assosciation in VLBW infants	8
Yau, G. S. K.	2016	Hong Kong	Retrospective review	Pre-eclampsia	N/A	N/A	0.26 (0.08–0.76)	GA, BW, gestational diabetes mellitus, *in-vitro* fertilization, postnatal hypotension, inotrope use, bronchopulmonary dysplasia (BPD), surfactant use, invasive mechanical ventilation, mean oxygen concentration, patent ductus arteriosus (PDA), NSAID use, anemia, blood transfusion, IVH, hypoglycemia	Protective	9
Gebeşçe, A.	2016	Istanbul, Turkey	Retrospective review	Maternal pre-eclampsia	48	162	3.200 (1.002–11.535)	BW, respiratory distress syndrome (RDS), ventilator treatment, BPD	Increased risk	7

N/A: not available.

**Table 2:** Meta-analysis of Association of gestational hypertensive disorders with ROP.

Type of explosure	No of Studies	Sample size	Overall effect			Heterogeneity		Egger’s
			OR (95%CI)	Z score	P Value	I^2,^%	Q (P)	
PET + Gestatioanl Hypertension*	18	45281	0.89(0.67–1.16)	0.88	0.38	81	<0.00001	0.688
PET + Gestational Hypertension^*,^‡	6	7369	0.89(0.66–1.20)	0.76	0.45	77	0.0007	0.089
PET*	15	37930	0.85(0.63–1.15)	1.06	0.29	81	<0.00001	0.919
Gestational Hypertension*	4	30739	1.10(0.89–1.36)	0.86	0.39	45	0.14	0.643
PET + Gestational Hypertension†	10	35960	1.35(0.87–2.08)	1.35	0.18	84	<0.00001	0.302
PET†	9	33875	1.29(0.81–2.04)	1.07	0.28	85	<0.00001	0.507
Gestational Hypertension†	2	27558	1.25(0.54–2.88)	0.53	0.60	76	0.04	NA

^*^Pooled unadjusted OR.

^‡^Outcome is severe ROP.

^†^Pooled adjusted OR.
