# Supplementary material for: Association of Gestational Hypertensive Disorders with Retinopathy of prematurity: A Systematic Review and Meta-analysis
Source: Sci Rep. 2016 Aug 5;6:30732. doi: 10.1038/srep30732 (PMC4974497; doi:10.1038/srep30732)
Supplement: Supplementary Information [file srep30732-s1.pdf]

Title: Association of Gestational Hypertensive Disorders with Retinopathy of prematurity: A Systematic Review and Meta-analysis

Author lists: Priscilla Y.L. Chan, Shu-Min Tang, Sunny C.L. Au, Shi-Song Rong, Henry H.W. Lau, Simon T.C. Ko, Danny S.C. Ng, Li Jia Chen, Jason C.S. Yam

Supplementary Table 1 Searching strategy

Supplementary Table1: Searching strategy in Medline and Embase

1.exp "eclampsia and preeclampsia"/ or exp preeclampsia/
2.preeclampsia/ or preeclampsia.mp.
3.exp pregnancy toxemia/
4.Edema Proteinuria Hypertension Gestosis.mp.
5.pregnancy toxemia.mp.
6.pregnancy toxemias.mp.
7.gestational hypertension.mp. or exp maternal hypertension/
8.1 or 2 or 3 or 4 or 5 or 6 or 7
9.Retinopathy of Prematurity.mp. or exp retrolental fibroplasia/
10.retrolental fibroplasia*.mp.
11.ROP.mp.
12.neonatal outcome.mp.
13.neurodevelopment outcome.mp.
14.preterm outcome.mp.
15.9 or 10 or 11 or 12 or 13 or 14
16.8 and 15

Supplementary Table 2. Checklist summarising compliance with MOOSE guidelines

Reporting background should include	
Problem definition	Yes
Hypothesis statement	Yes
Description	Yes
Type of exposure or intervention used	Yes
Type of study designs used	Yes
Study population	Yes
Reporting of search strategy should include	
Qualifications of searches (e.g. librarians and investigators)	Yes
Search strategy, including time period included in the synthesis and keywords	Yes
Effort to include all available studies, including contact with authors	Yes
Databases and registries searched	Yes
Search software used, name and version, including special features	Yes
Use of hand searching (e.g. reference lists of obtained articles)	Yes
List of citations located and those excluded including justification	Available on request
Method of addressing articles published in languages other than English	Yes
Method of handling abstracts and unpublished studies	Yes
Description of any contact with authors	No
Description of relevance or appropriateness of studies assembled for assessing the hypothesis to be tested	Yes
Rationale for the selection and coding of data (eg, sound clinical principles or convenience)	Yes
Documentation of how data were classified and coded (eg, multiple raters, blinding, and interrater reliability)	Yes
Assessment of confounding (eg, comparability of cases and controls in studies where appropriate)	Yes
Assessment of study quality, including blinding of quality assessors; stratification or regression on possible predictors of study results	Yes
Assessment of heterogeneity	Yes
Description of statistical methods (eg, complete description of fixed or random effects models, justification of whether the chosen models account for predictors of study results, dose-response models, or cumulative meta-analysis) in sufficient detail to be replicated	Yes
Provision of appropriate tables and graphics	Yes
Graphic summarizing individual study estimates and overall estimate	Yes
Table giving descriptive information for each study included	Yes
Results of sensitivity testing (eg, subgroup analysis)	Yes
Indication of statistical uncertainty of findings	Yes
Reporting of discussion should include	
Quantitative assessment of bias (eg, publication bias)	Yes
Justification for exclusion (eg, exclusion of non–English-language citations)	Yes
Assessment of quality of included studies	Yes
Reporting of conclusions should include	
Consideration of alternative explanations for observed results	Yes
Generalization of the conclusions (ie, appropriate for the data presented and within the domain of the literature review)	Yes
Guidelines for future research	Yes
Disclosure of funding source	Yes
